# Effect of iron fortification on anaemia and risk of malaria among Ghanaian pre-school children with haemoglobinopathies and different ABO blood groups

**DOI:** 10.1186/s40795-023-00709-w

**Published:** 2023-03-23

**Authors:** Samuel Kofi Tchum, Samuel Asamoah Sakyi, Fareed Arthur, Bright Adu, Latifatu Alhassan Abubakar, Felix Boakye Oppong, Francis Dzabeng, Benjamin Amoani, Thomas Gyan, Kwaku Poku Asante

**Affiliations:** 1 Department of Biochemistry and Biotechnology, College of Sciences, Kwame Nkrumah University of Science and Technology, Kumasi, Ghana; 2 Kintampo Health Research Centre, Ghana Health Service, Kintampo-North, Bono East Region Ghana; 3 Department of Molecular Medicine, School of Medical Sciences, Kwame Nkrumah University of Science and Technology, Kumasi, Ghana; 4 Department of Immunology, Noguchi Memorial Institute for Medical Research, College of Health Sciences, University of Ghana, Legon, Accra, Ghana; 5 West African Centre for Cell Biology of Infectious Pathogens, University of Ghana, Accra, Ghana; 6 Department of Biomedical Sciences, School of Allied Health Sciences, University of Cape Coast, Cape Coast, Ghana

**Keywords:** Polymorphisms, Haemoglobinopathies, Anaemia, Malaria, Iron fortification

## Abstract

**Background:**

Haemoglobinopathies such as sickle cell disorder and glucose-6-phosphate dehydrogenase (G6PD) deficiency as well as differences in ABO blood groups have been shown to influence the risk of malaria and/or anaemia in malaria-endemic areas. This study assessed the effect of adding MNP containing iron to home-made weaning meals on anaemia and the risk of malaria in Ghanaian pre-school children with haemoglobinopathies and different ABO blood groups.

**Methods:**

This study was a double-blind, randomly clustered trial conducted within six months among infants and young children aged 6 to 35 months in rural Ghana (775 clusters, *n* = 860). Participants were randomly selected into clusters to receive daily semiliquid home-prepared meals mixed with either micronutrient powder without iron (noniron group) or with iron (iron group; 12.5 mg of iron daily) for 5 months. Malaria infection was detected by microscopy, blood haemoglobin (Hb) levels were measured with a HemoCue Hb analyzer, the reversed ABO blood grouping microtube assay was performed, and genotyping was performed by PCR–RFLP analysis.

**Results:**

The prevalence of G6PD deficiency among the study participants was 11.2%. However, the prevalence of G6PD deficiency in hemizygous males (8.5%) was significantly higher than that in homozygous females (2.7%) (*p* = 0.005). The prevalence rates of sickle cell traits (HbAS and HbSC) and sickle cell disorder (HbSS) were 17.5% and 0.5%, respectively. Blood group O was dominant (41.4%), followed by blood group A (29.6%) and blood group B (23.3%), while blood group AB (5.7%) had the least frequency among the study participants. We observed that children on an iron supplement with HbAS had significantly moderate anaemia at the endline (EL) compared to the baseline level (BL) (*p* = 0.004). However, subjects with HbAS and HbAC and blood groups A and O in the iron group had a significantly increased number of malaria episodes at EL than at BL (*p* < 0.05). Furthermore, children in the iron group with HbSS (*p* < 0.001) and the noniron group with HbCC (*p* = 0.010) were significantly less likely to develop malaria.

**Conclusions:**

Iron supplementation increased anaemia in children with HbAS genotypes and provided less protection against malaria in children with HbAC and AS and blood groups A and O.

**Trial registration:**

clinicaltrials.gov Identifier: NCT01001871. Registered 27/10/2009.

Registration number: https://clinicaltrials.gov/ct2/show/record/NCT01001871.

## Introduction

Haemoglobinopathies and ABO blood types have been shown to influence the risk of severe malaria and anaemia [1–3]. It has been proposed that in haemoglobin S (HbS) and C (HbC), a reduction in parasite growth during tissue hypoxia together with lowered cytoadherence due to poor display of *Plasmodium falciparum* erythrocyte membrane protein-1 (*Pf*EMP1) enhances parasite clearance, which reduces the risk of clinical malaria [4–6]. Moreover, deficiency of glucose-6-phosphate dehydrogenase (G6PDD) has been reported to decrease malaria parasitaemia [7, 8]. Genetic and pathogenic mechanisms have shown that blood group O children are more resistant to malaria than other blood types, while blood group A is detrimental to malaria [9–11].

Over the past two decades, iron (Fe) intervention studies in many parts of the world, including Ghana, have shown that the use of microencapsulated iron micronutrient powder (MNP) “Sprinkles” (minerals and vitamins) is cheaper, safer because it is cost effective and has less side effects compared to the use of iron droplets or syrups, and more effective in reducing anaemia rates by up to 60% [12–21]. Moreover, long-term use of iron-fortified MNP did not increase the incidence of malaria among infants and young children living in high malaria burden areas when appropriate anti-malaria therapy and insecticide-treated bed nets were provided [22].

Nonetheless, there is limited knowledge about the effects of iron-fortified micronutrient diets on ABO blood groups, haemoglobin and G6PD genotypes and the risk of contracting malaria and anaemia among pre-school children living in malaria endemic areas. Furthermore, the geographical distribution of ABO blood groups, haemoglobin and G6PD genotypes are often associated with anaemia and malaria protection among certain ethnic groups, but their effect among iron-fortified infants and young children is unknown [12, 16, 23]. Against this background, we present a secondary analysis of double-blind, cluster-randomized trial data [22] aimed at determining the effect of ABO blood groups, haemoglobin and G6PD genotypes against the risk of malaria and anaemia among Ghanaian infants and young children receiving iron-containing MNP in high malaria burden areas.

## Materials and methods

### Study design

This retrospective community-based, double-blinded, cluster-randomized controlled trial (RCT) was conducted in 2009 among children aged 6 to 35 months over a 6-month period in a rural setting in the middle belt of Ghana [22]. The children were enrolled and randomized into clusters to receive MNP with iron (intervention group) or without iron (placebo group). The MNP was distributed weekly and advised to be mixed with a home-prepared weaning meal for five months followed by a further month without micronutrient powder. Insecticide-treated bed nets were provided during enrollment, as well as malaria treatment when required.

### Study site

This study was conducted in Wenchi Municipal and Tain District located within the forest-savanna zone in the middle belt of Ghana. The mean temperature in the area ranges between 18 and 38 °C with an average rainfall of 1250 mm per annum, occurring mainly between May and October, as previously reported elsewhere [22]. Malaria is holoendemic in Ghana, with an estimated 3.2 million cases, mostly caused by *Plasmodium falciparum* [24]. At the study site, malaria transmission is highest during the rainy season (April-November) [22, 25], and the prevalence of anaemia among preschool-aged children is 76.1% (95% CI, 73.9% -78.2%) [26]. In 2010, the population of Wenchi and Tain was 153,633, with 11,215 children younger than five years, representing approximately 0.3% of the total preschool-aged child population in Ghana [22]. There are 99 smaller communities across the municipality and district, consisting of 8,548 compounds [22]. A compound was considered eligible for inclusion in the study if the resident families had at least one child younger than 35 months but older than five months. However, all compounds and households in the municipality and district had been previously enumerated into a geographical information system (GIS) database and were used to identify targeted study communities as previously reported elsewhere [22].

### Study population

Approximately 2220 infants and young children were screened. Those on weaning foods in addition to breastmilk, free from major illness, afebrile and living in the study area for the duration of the trial were enrolled in the study. Parental consent was obtained after the possible risks and benefits had been discussed with the parents or guardians (caregivers of the eligible child) in the appropriate local language and signed. Therefore, in 2013, the nutritional status was defined by WHO as follows; Moderate wasting (MW): -3 ≤ weight-for-height (WLZ/WHZ) < -2: Moderate stunting (MS): -3 < length-for-height (LAZ/HAZ) < -2: if height or length was measured differently according to the age: Moderate underweight (MUW): -3 ≤ weight-for-age (WAZ) < -2 as previously described in details elsewhere [16, 22, 27, 28]. The exclusion criteria included severe anaemia (haemoglobin < 70 g/L); WHZ < -3 (severe wasting), and kwashiorkor (defined as evidence of oedema); IDA; congenital abnormality or any chronic illness; and receipt of iron supplements within the past six months. However, severely anaemic and malnourished children were referred to the local healthcare facility for treatment according to Ghana Health Service guidelines [22, 29, 30].

### Recruitment and randomization

A detailed overview of the context of trial recruitment and randomization has previously been published elsewhere [22]. Subjects were recruited from selected communities in Wenchi Municipal and Tain District once permission was obtained from the opinion elders, as well as the caregivers. A powdered mineral and vitamin fortificant product, “Sprinkles” (Ped-Med Limited and Sprinkles Global Health Initiative Inc. India) was used in the study. The World.

Health Organization (WHO), International Nutritional Anemia Consultative Group (INACG), and United Nations Children's Fund (UNICEF) recommended that the ‘Sprinkle’s formulation should contain 12.5 mg of elemental iron (as microencapsulated ferrous fumarate) plus ascorbic.acid (30 mg), vitamin A (400 μg), and zinc (5 mg) [31, 32]. The placebo group received a similar MNP without iron. The caregivers were instructed to mix MNP with a small portion of a semi-liquid food, such as porridge or thin gruel, and feed it to the child every day for five months, after which the dosing regimen was discontinued for an additional month, as published elsewhere [22, 23]. Detailed procedures on the consumption of MNP with the meal by the participants including the estimated actual amount of iron intake have been previously reported elsewhere[16, 22, 23].

### Data and specimen collection

Baseline (BL) and endline (EL) MNP intervention of the child’s health was assessed (including axillary temperature), and a capillary blood sample of 500µL from the finger or heel was taken into a 0.5 mL ethylene diamine tetra-acetic acid (EDTA) tube. A HemoCue Hb 201^+^ analyzer (HemoCue AB, Angelholm, Sweden) was used to measure haemoglobin levels, and children found to be severely anaemic (Hb < 70 g/L) were immediately referred. The Malaria Rapid Diagnostic Test (RDT) (Paracheck *Pf* Unit, Orchid Biomedical Systems Verna, Goa, India) was performed quickly in the field, and a 3-day course of treatment was given to those who tested positive for malaria. They were only enrolled later upon recovery if any other eligibility requirements had been met. The remaining sample was transferred to the haematological, malaria microscopy and immunogenetic testing laboratory.

During the MNP intervention period and at six-month follow-up, children found to be febrile (i.e., axillary temperature > 37.5 °C) or have had a fever in the past 48 h were, blood sampled of 100μL in 0.5 mL EDTA for haematological and malaria tests. Anthropometric, socioeconomic, demographic, adherence to MNP intervention, insecticide-treated bed net use and participants' morbidity data were collected during the study as previously described in details elsewhere [22, 23].

### Processing and analysis of the specimen

Both thick and thin smears were prepared on the same slide at the laboratory for malaria parasitaemia and speciation. Thin films were fixed with methanol, and both smears were stained with Giemsa. Two independent microscopists read each slide, while a third microscopy was conducted in case of over 50% discrepancy as previously described [22, 23]. Complete blood cell count (CBC) were determined using a haematology autoanalyzer (Horiba ABX Micros 60-OT-CT-OS-CS, France) and an automated microplate reader (DYNEX Technologies, Inc. USA) for matrix ABO reverse grouping was used to determine the blood groups of participants [33]**.** Human genomic DNA was purified from blood samples in EDTA tubes stored between 2 and 8 °C using the QIAamp® DNA Mini Kit (QIAGEN Sample and Assay Technologies, USA) protocol based on published methods [34, 35].

Polymerase chain reaction (PCR) for haemoglobin genotyping amplified a 358 base pair region within the β-globin gene in human genomic DNA using primer sequences SC1F: 5’-AGGAGCAGGGAGGGCAGGA-3’ and SC2R: 5’-TCCAAGGGTAGACCACCAGC-3’ based on published methods [36]. PCR amplification was performed in a 50μL reaction containing 20 – 40 ng genomic DNA, 20 mM of each primer (DNA Technology A/S, Risskov, Denmark), 25 mM of each of the four deoxynucleotide triphosphates (dNTPs), 5.0 units of ProofStart DNA Polymerase (QIAGEN Ltd. UK and Ireland) and 15 mM MgCl_2_ (QIAGEN Sample and Assay Technologies, USA) and 10X ProofStart PCR Buffer (QIAGEN Sample and Assay Technologies, USA) containing 15 mM MgCl_2_. The cycling conditions were as follows: initial denaturation at 95 °C for 15 min, 34 cycles of denaturation at 95 °C for 45 s, annealing at 65 °C for 45 s and extension at 72 °C for 120 s, followed by a final extension at 72 °C for 10 min in a Peltier thermal cycler (PTC) – 200 model (M. J Research Inc; MA, USA). The PCR product was analyzed by electrophoresis (Apelex Power station, France) at 90 V for 60 min using 1µL of blue DNA loading dye (Promega Co, USA) on 3% (w/v) agarose (SeaKem® GTG® Agarose, Lonza, Rockland, ME, USA) with ethidium bromide (AppliChem, Damstadt, Germany) staining, and pictures were taken using the Ultra-Violet Product (UVP) Biospectrum AC Imaging system with Biochemi-camera (AH Diagnostics, Cambridge, UK).

Thereafter, restriction fragment length polymorphism (RFLP) was used to differentiate between the point mutations (which translate into the various sicklings) within the amplified gene region. The enzymes *Mnl*I and *Dde*I (New England BioLabs Inc. Ipswich, MA, USA) were added to the two separate PCR products and incubated at 37 °C for 3 h and run on 3% agarose gel electrophoresis for 45 min to further differentiate into their various genotypes [36].

Polymerase chain reaction for G6PD genotyping amplified 354 base pair (allele 202 polymorphism detection) and 296 base pair (allele 376 polymorphism detection) DNA regions within the G6PD gene in human genomic DNA using primer sequences 5’-CAG CCA CTT CTA ACC ACA CAC CT-3′5’-CCG AAG TTG GCC ATG CTG GG-3’ for allele 202 (354 base pairs) and 5’-CTG TCT GTG TGT CTG TCT GTC C-3’’5’-GGC CAG CCT GGC AGG CGG GAA GG-3’ for allele 376 (296 base pairs) manufactured by Sigma-Aldrich Inc. PCR amplification was performed in a 50μL reaction containing 20 – 40 ng genomic DNA, 20 mM of each primer (DNA Technology A/S, Risskov, Denmark), 25 mM of each of the four deoxynucleotide triphosphates (dNTPs), 5.0 units of ProofStart DNA Polymerase (QIAGEN Ltd. UK and Ireland) and 10X ProofStart PCR Buffer (QIAGEN) containing 15 mM MgCl_2_.

The cycling conditions were as follows: initial denaturation at 95 °C for 10 min, 34 cycles of denaturation at 95 °C for 30 s, annealing at 60 °C for 30 s and extension at 72 °C for 60 s, followed by a final extension at 72 °C for 5 min in a PTC – 200. The PCR product was analyzed by electrophoresis on 3% (w/v) agarose with ethidium bromide staining, and pictures were taken using UVP. The PCR products were digested with restriction enzymes *Nla*III and FokI (New England BioLabs Inc.) and incubated at 37 °C for 3 h. Thereafter, 3% agarose gel electrophoresis was run for 1 h. The restriction enzyme *Nla*III cleaved allele 202 PCR product (354 base pairs), while the *Fok*I restriction enzyme cleaved allele 376 (296 base pairs) to enable discrimination between the various polymorphisms [37–40].

### Ethics approvals

The study protocol was reviewed and approved by the ethics committees of Kintampo Health Research Centre Institutional Ethics Committee; Approval ID: KHRC/IEC/FEA/2009–2, Ghana Health Service Ethics Committee, Food and Drug Authority of Ghana and Hospital for Sick Children Research Ethics Board (REB), Toronto, and Canada REB File No.: 1000013476 approved the study as previously reported in detail elsewhere [22, 23]. This study was appropriately registered: https://clinicaltrials.gov/ct2/show/record/NCT01001871.

All methods were performed in accordance with the relevant guidelines and regulations by the Declaration of Helsinki. The trial was overseen by an independent Data and Safety Monitoring Board (DSMB). International and local health policymakers with experience in randomized controlled trials, nutrition, paediatrics, statistics, and social sciences made up the members of the DSMB. At the end of the recruiting phase and halfway through the intervention stage, the DSMB's statistician summarized the accumulated outcome data for any major adverse effects. The primary caregivers gave their consent for their children to participate in this study. For the interim analysis, if the iron group had any more major adverse events (i.e., hospital admissions or deaths) than the noniron group, the trial would be terminated.

### Statistical analysis

Clinical and epidemiological data were entered into a data processing system for Visual Fox Pro version 9.0 (Microsoft Corporation, Redmond, WA. USA), imported into STATA version 14.0 (Statcorp Inc. TX. USA) and SigmaPlot version 11.0 (Systat Software Inc. CA. USA) for analysis. World Health Organization anthropometric software was used to transform the age, weight and length of the study participants into growth indices: weight-for-age, weight-for-length and length-for-age z-scores. Infants with z-scores less than -2 standard deviations of the median reference length-for-age (LAZ), weight-for-length (WLZ) and weight-for-age (WAZ) were classified as stunted, wasted and underweight, respectively [22]. An overall wealth index was computed for each study participant using principal component analysis [41, 42] by including the number and type of assets (such as televisions, cars, electricity, toilet facilities, house ownership) available in a study participant’s household. Study participants were grouped by their wealth indices into high or low socioeconomic status. The distribution and comparison of proportions of the various haemoglobinopathies and polymorphisms were analyzed using the chi-square and Fisher’s exact tests. Multiple logistic regression analysis was used to determine whether haemoglobinopathies and polymorphisms were associated with the risk of contracting malaria and anaemia in the cohort study. Multicollinearity was checked using variance to inflation ratio and these were well below 10 and the tolerance statistics were all above 0.2. The model goodness-fit-test was done using log-likelihood chi-square test. A *p*-value < 0.05 was considered statistically significant.

## Results

### Baseline characteristics of the study participants

**Fig. 1:**
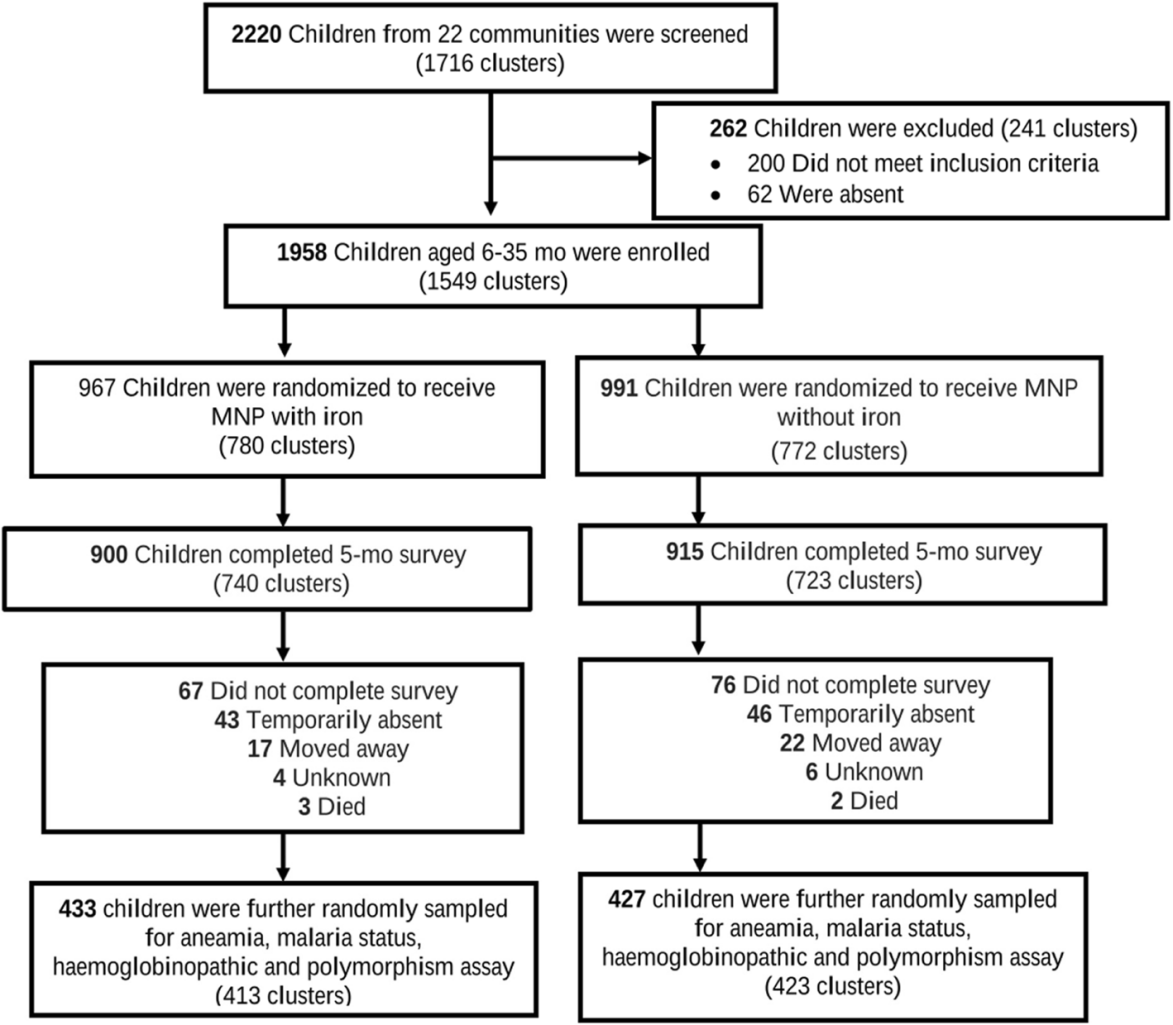
Schematic representation of study participants. Micronutrient powder (MNP), month (mo), a cluster is an identified compound with 1 or more households (or family units) residing together with at least 1 eligible child recruited into the study

A total of 2220 children were screened from 22 communities and over 80% were recruited to receive the multi-micronutrient powder (Fig. 1). This study used data and samples from a main study conducted to see how providing a micronutrient powder (MNP) with or without iron affected the incidence of malaria in pre-school children living in a malaria-endemic region [22]. The remaining 860 children from 775 clusters were randomized into 433 and 427 children from the iron and noniron groups respectively, which accounted for about 44% of the recruited population in order to meet the sample size requirements (Fig. 1).

Baseline anthropometric, socioeconomic and demographic characteristics were similar between the two groups (*p* > 0.05) (Table 1). Of 409 children in the iron group, the baseline asymptomatic malaria parasitaemia prevalence was 25.9%, and 28.9% of 422 were in the noniron group. (Additionally, at baseline, moderate anaemia was observed in 40.4% of 431 children in the iron group and 35.7% of 434 in the noniron group (Table 1).

**Table 1:** Baseline characteristics of study participants

Characteristics	Iron Group (*n* = 433)	Noniron Group (*n* = 427)	*p*-values
**Age, mean (SD) [range], mo**	19.7 (8.5) [6–35]	19.3 (8.6) [6–35]	0.74
**Gender (%), Male: female**	52:48	51:49	0.97
**Anthropometric status**			0.55
Wasting, n (%) [95% CI]	40 (9.2) [7.3–11.0]	32 (7.4) [5.7–9.1]	
Weight-for-length Z score mean (SD)	-0.62 (1.0)	-0.59 (1.0)	
Stunted growth, n (%) [95% CI]	64 (14.7) [12.4–17.1]	60 (13.8) [11.5–16.1]	
Length-for-age Z score, mean (SD)	-0.81 (1.5)	-0.80 (1.2)	
Underweight, n (%) [95% CI]	56 (12.9) [10.7–15.4]	46 (10.6) [8.5–12.6]	
Weight-for-age Z score mean (SD)	-0.87 (1.2)	-0.85 (1.0)	
**Prevalence of asymptomatic malaria parasitaemia, n (%)**	409 (23.0)	422 (25.4)	0.47
**Prevalence of moderate anaemia, n (%)**	433 (40.4)	427 (35.7)	0.16
**Bednet use, n (%)**	433 (100)	427 (100)	
Yes	379 (87.5)	380 (89.0)	
No	34 (8.2)	43 (10.1)	
Missing	20 (4.6)	4 (0.9)	
**Parasitaemia, n, geometric mean, count/µL of blood**	409, 2868.2	422, 2478.6	0.63
**Household education, n (%)**	433 (100)	427 (100)	0.19
None	138 (31.9)	137 (32.1)	
Primary school	89 (20.6)	64 (15.0)	
Middle/continuation, JSS school	161 (37.2)	185 (43.3)	
Technical, commercial, SSS, secondary school	23 (5.6)	33 (7.7)	
Tertiary institution	2 (0.5)	4 (0.9)	
Missing	20 (4.6)	4 (0.9)	
**No. of people in household, n, mean (SD)**	433, 6.0 (2.8)	427, 6.2 (5.3)	0.52
**Wealth index of household, n (%)**	433 (100)	427 (100)	0.11
High	125 (28.9)	142 (33.2)	
Low	288 (66.5)	278 (65.0)	
Missing	20 (4.6)	4 (0.8)	

Frequency (n) and percentages (%) of participants, 95% confidence interval (95% CI), standard deviation (SD), age in months (mo), estimated using the World Health Organization growth charts (-2 SD), two-sample Wilcoxon rank-sum (Mann–Whitney) and 1-sided Fisher's exact tests for *p*-values

### Prevalence of ABO blood groups and haemoglobinopathies amongst participants

The prevalence of G6PD deficiency among the study participants was 11.2%. However, the prevalence of female homozygous G6PDD was 2.7%, whereas that of male hemizygous G6PDD was 8.5% and the deficiency was statistically significant between both sexes (*p* = 0.005). The sickling traits (HbAS & HbSC) and disorder (HbSS) among participants were 17.5% and 0.5%, respectively (Table 2). The haemoglobin SS (HbSS) genotype frequency was the lowest (0.5%). Blood group O (41.4%) was dominant, while blood group AB (5.7%) was the rarest. Children with blood group A were more prevalent than those with blood group B (Table 2).

**Table 2:** Prevalence of hemoglobinopathies and ABO blood groups

**G6PD genotypes**	**A-A-**		**AA**		**AB**	**BB**	**Total**
n (%)	96 (11.2)		236 (27.6)		95 (11.1)	430 (50.1)	857 (100)
Male: female (%)	8.5:2.7		14.3:13.3		0:11.1	28.1:22.0	51:49
*P*-values	0.005		0.919		NA	0.668	0.972
**Hb genotypes**	**Hb AA**	**Hb AC**	**Hb CC**	**Hb AS**	**Hb SC**	**Hb SS**	**Total**
n (%)	627 (72.9)	63 (7.3)	15 (1.7)	106 (12.3)	45 (5.2)	4 (0.5)	860(100)
**ABO Blood groups**	**A**	**B**		**AB**	**O**		**Total**
n (%)	244 (29.6)	192 (23.3)		47 (5.7)	341 (41.4)		824 (100)

Data reported as the number (n) and percentage (%) of participants, glucose-6-phosphate dehydrogenase (G6PD). For G6PD genotype: male hemizygous (deficient variant or mutant) = A-; male (non-deficient variant) = A; male (wild type) = B; female homozygous (deficient variant or mutant) = A-/A-; female heterozygous (intermediate type) = A/B; and female homozygous (non-deficient variant) = A/A; female homozygous (wild type) = B/B. Haemoglobin (Hb); For Hb genotype: sickling traits (HbAS & HbSC), sickle disorder (HbSS), haemoglobin C traits (HbAC), normal or wild-type haemoglobin (HbAA), variant haemoglobin (HbCC) and 1-sided Fisher's exact tests for *p*-values

### Risk of anaemia and malaria on host genes

Multivariate analysis showed that blood group AB children (aOR 2.24, 95% CI 1.03 – 4.85, *p* = 0.041) were significantly more likely to develop anaemia than blood group A children. However, ABO blood group were not statistically associated with malaria (Table 3). The result also shows that compared to children with HbAA, the children with HbCC (aOR: 0.7, 95% CI: 0.23 – 2.23, *p* > 0.05) were not significantly less likely to develop malaria (Table 3). Furthermore, children with G6PD genotype AA were less likely to develop malaria compared to A-A- (aOR: 0.56, 95% CI: 0.32—0.96, *p* < 0.05). Conversely, no significant association were found between G6PD genotypes and anaemia (Table 3).

**Table 3:** Multiple logistic regression analysis of the association between selected host genes and anaemia and malaria

	**Anaemia**		**Malaria**	
**Host characteristics**	**aOR (95% CI)**^a^	***P*-value**	**aOR (95% CI)**^a^	***P*-value**
**Experimental group**				
No iron	1		1	
Iron	0.85 (0.63-1.15)	0.303	0.95 (0.71-1.26)	0.708
**G6PD genotypes**				
A-A-	1		1	
AA	0.87 (0.51-1.50)	0.622	0.56 (0.32-0.96)	0.035
AB	0.85 (0.45-1.59)	0.605	0.68 (0.37-1.27)	0.230
BB	0.76 (0.46-1.26)	0.291	0.49 (0.30-0.82)	0.006
**Hb genotypes**				
AA	1		1	
AC	1.04 (0.58-1.86)	0.885	0.61 (0.35-1.07)	0.083
AS	1.37 (0.86-2.20)	0.185	1.58 (0.99-2.51)	0.054
CC	1.27 (0.36-4.49)	0.714	0.71 (0.23-2.23)	0.561
SC	1.07 (0.57-2.01)	0.831	1.02 (0.56-1.89)	0.940
SS	-		-	-
**ABO group**				
A	1		1	
AB	2.24 (1.03-4.85)	0.041	0.79 (0.42-1.51)	0.482
B	1.02 (0.68-1.54)	0.922	1.23 (0.82-1.85)	0.315
O	1.02 (0.71-1.45)	0.932	0.99 (0.70-1.39)	0.929

Data reported as 95% confidence interval (95% CI), percentage (%) of participants, glucose-6-phosphate dehydrogenase (G6PD). For G6PD genotype: deficient variant or mutant = A-A-, non-deficient variant = AA, wild type = BB, intermediate type = AB, Haemoglobin (Hb), For Hb genotype: sickling traits (HbAS & HbSC), sickle disorder (HbSS), haemoglobin C traits (HbAC), normal or wild-type haemoglobin (HbAA), variant haemoglobin (HbCC)

^a^Adjusted for child’s age and sex

### Effect of ABO blood groups and haemoglobinopathies on anaemia

The children from the iron group with HbAS had significantly higher moderate anaemia at EL than at BL (*p* = 0.004) (Table 4). However, G6PD and blood grouping did not influence the anaemic status of the children receiving iron at EL compared to children at BL (Table 4).

**Table 4:** Effect ABO blood groups and haemoglobinopathies on anaemia

**Prevalence of Anaemia n (%)**													
	**Iron group**				**Noniron group**				**Endline**				
	**Baseline**	**Endline**		***p*-values**	**Baseline**	**Endline**		***p*-values**	**Iron group**		**Noniron group**		***P*-value**
**Type of Anaemia**	**Mod**	**Mod**	**Sev**		**Mod**	**Mod**	**Sev**		**Mod**	**Sev**	**Mod**	**Sev**	
**Haemoglobin genotype**													
**AA**	128(30.0)	178(41.3)	19(4.4)	0.014	104(24.4)	158(37.0)	32(7.4)	< 0.001	178(41.3)	19(4.4)	158(37.0)	32(7.4)	0.910
**AC**	10(2.3)	16(3.7)	2(0.4)	0.310	13(3.0)	20(4.7)	1(0.2)	0.006	16(3.7)	2(0.4)	20(4.7)	1(0.2)	0.870
**AS**	**23(5.4)**	**30(7.0)**	**3(0.7)**	**0.004**	24(5.6)	33(7.7)	6(1.4)	0.150	30(7.0)	3(0.7)	33(7.7)	6(1.4)	0.850
**CC**	3(0.7)	3(0.7)	0(0.0)	0.700	2(0.4)	4(0.9)	1(0.2)	1.00	3(0.7)	0(0.0)	4(0.9)	1(0.2)	0.580
**SC**	6(1.4)	11(2.5)	1(0.2)	0.190	8(1.8)	11(2.6)	4(0.9)	0.130	11(2.5)	1(0.2)	11(2.6)	4(0.9)	0.550
**SS**	1(0.2)	0(0.0)	1(0.2)	NA	1(0.5)	1(0.2)	0(0.0)	0.670	0(0.0)	1(0.2)	2(0.4)	0(0.0)	0.640
**G6PD genotypes**													
A-A-	19(4.5)	24(5.6)	2(0.5)	0.770	25(5.9)	28(6.6)	8(1.9)	0.063	24(8.0)	2(0.5)	28(6.6)	8(1.9)	0.520
AA	47(11.1)	71(16.6)	9(2.1)	0.250	35 (8.2)	60(14.1)	9(2.1)	0.810	71(16.6)	9(2.1)	60(14.1)	9(2.1)	0.730
AB	26(6.1)	41(9.6)	6(1.4)	0.003	73(17.1)	44(10.3)	5(1.2)	< 0.001	41(9.6)	6(1.4)	44(10.3)	5(1.2)	0.310
BB	78(18.4)	100(23.4)	9(2.1)	0.560	190(44.6)	96(22.5)	22(5.2)	0.052	100(23.4)	9(2.1)	96(22.5)	22(5.2)	0.420
**ABO phenotype**													
**A**	51(12.5)	67(16.3)	5(1.2)	< 0.001	38(9.3)	72(17.6)	11(2.7)	0.001	67(16.3)	5(1.2)	72(17.6)	11(2.7)	0.320
**AB**	12(3.0)	20(4.9)	3(0.7)	0.150	5(1.2)	10(2.4)	3(0.7)	0.230	20(4.9)	3(0.7)	10(2.4)	3(0.7)	0.190
**B**	32(7.9)	41(10.0)	6(1.5)	< 0.001	38(9.2)	55(13.3)	13(3.2)	0.005	41(10.0)	6(1.5)	55(13.3)	13(3.2)	0.340
**O**	71(17.4)	102(24.8)	12(2.9)	0.590	66(16.0)	87(21.1)	16(3.9)	0.009	102(24.8)	12(2.9)	87(21.1)	16(3.9)	0.360

Data reported as moderate (Mod.), severe (Sev.), frequency (n), percentage (%) of participants, glucose-6-phosphate dehydrogenase (G6PD), ABO blood groups (ABO), For G6PD genotype: deficient variant or mutant = A-A-, non-deficient variant = AA, wild type = BB, intermediate type = AB, Haemoglobin (Hb), For Hb genotype: sickling traits (HbAS & HbSC), sickle disorder (HbSS), haemoglobin C traits (HbAC), normal or wild-type haemoglobin (HbAA), variant haemoglobin (HbCC) and *p*-values were a 1-sided Fisher's exact test

### Effect of haemoglobinopathies and ABO blood groups on clinical malaria

Drawing on the analysis in Table 5, a clinical malaria episode is defined as fever (temperature more than 37.5 °C) or a history of fever in the last 24 h with malaria parasitaemia more than 5,000 counts/mL of blood [23]. The highest number of clinical malaria episodes experienced by a study participant following the intervention with iron was 5, while those without iron were 4 (Table 5). Participants with HbAS and AC alleles in the iron group had a significantly higher mean number of malaria episodes than children in the noniron group (*p* < 0.05). The mean number of malaria episodes among participants with blood groups A and O in the iron group was also significantly increased compared with children in the noniron group (*p* < 0.05) (Table 5). The iron group with the heterozygote G6PD AB had a significantly higher geometric mean parasitaemia 27,970 (95% CI, 11,498 – 68,041; 25/435) than children in the noniron group 11,729 (95% CI, 5745 – 23,946; 28/436) (*p* = 0.03) (not shown in Table 5).

**Table 5:** Effect of host genes on clinical malaria episodes

**Number of participants, mean episodes (ranges)**											
**Haemoglobin genotype**	**Iron Gp**	**Noniron Gp**	***p*-values**	**G6PD Genotype**	**Iron Gp**	**Noniron Gp**	***p*-values**	**ABO phenotype**	**Iron Gp**	**Noniron Gp**	***p*-values**
**AA**	325,0.9(0–5)	302,0.9(0–4)	0.3	**A-A-**	45,1.6(0–5)	51,1.6(0–5)	0.637	**A**	**122,0.9(0–4)**	**122,0.7(0–4)**	** < 0.001**
**AC**	**29,0.8(0–5)**	**34,0.8(0–3)**	**0.005**	**AA**	123,1.3(0–5)	113,1.1(0–5)	0.065	**AB**	28,0.9(0–5)	19,0.7(0–3)	0.1
**AS**	**49,1.2(0–4)**	**57,0.7(0–4)**	**0.005**	**AB**	78,1.4(0–6)	73,1.4(0–5)	0.795	**B**	86,0.9(0–5)	106,0.9(0–4)	0.2
**CC**	5,0.8(0–3)	10,0.4(0–2)	0.4	**BB**	183,1.2(0–6)	191,1.2(0–5)	0.564	**O**	**175,0.8(0–5)**	**166,0.9(0–4)**	**0.001**
**SC**	23,0.6(0–4)	22,1.1(0–4)	0.6								
**SS**	1,1.0(1–1)	3,1.0(0–2)	0.09								

Data reported as the number of participants (n), mean malaria episodes (f), group (Gp), glucose-6-phosphate dehydrogenase (G6PD), ABO blood groups (ABO); For G6PD genotype: deficient variant or mutant = A-A-, non-deficient variant = AA, wild type = BB, intermediate type = AB; Haemoglobin (Hb), For Hb genotype: sickling traits (HbAS & HbSC), sickle disorder (HbSS), haemoglobin C traits (HbAC), normal or wild-type haemoglobin (HbAA), variant haemoglobin (HbCC) and *p*-values were a two-sample Wilcoxon rank-sum (Mann–Whitney)

## Discussion

The overall effect of home fortification improved the participants' overall Hb levels and iron status after the intervention, as previously reported in detail elsewhere [16, 22, 23]. Furthermore, when participants were provided with insecticide-treated nets to prevent malaria and appropriate antimalarial treatments to manage malaria infection, the home fortification effect did not increase their risk of malaria, as described in detail elsewhere [16, 22, 23]. Persistent holoendemic malaria conditions have led to the emergence of host genes that offer different effects against malaria and anaemia, but awareness of the risks or benefits they provide in large-scale iron intervention studies among infants and young children is poorly understood.

In our study, the basic characteristics of the participants in the study arms were homogeneous, and these values were in line with the studies of Zlotkin et al*.* [22]. A prevalence of G6PD deficiency of 11.2% was observed, and this was comparable to Carter et al*.* [43], studies in Burkina Faso (9.4%), Kenya (11.4, 13.5%) [43], and Ghana (13.4%) [43] and George et al*.* [44], a study in Nigeria (14.6%). However, the observed G6PDD prevalence rates from our study are higher than those of Carter et al*.* [43], a study in Mali (5.6% and 8.7%) and Tanzania (7.6%) [43], including Ghana (5.3%, 6.0%) [43, 45]. These variations in the prevalence rates suggest that the genes, although rare, are highly polymorphic (almost 500 variants), and clinically relevant variants may be ignored during G6PD genotyping in holoendemic malaria zones [46, 47].

The G6PDD prevalence among male children (8.5%) was significantly higher than that among females (2.7%) among the study participants. Similarly, Carter et al*.* [43] reported 8.9% deficiency among males and 4.5% among females in a similar geopolitical setting in Ghana [43]. This may explain why the recessive X-linked gene is consistent with G6PDD and more prevalent in males [48], with variable prevalence rates from one geopolitical zone to another among different ethnic communities [49]. The prevalence of haemoglobin trait (HbAS and HbSC) was 17.5%, and sickle cell disorder (SCD) (HbSS) was 0.5%; these findings were similar to studies in three countries (Nigeria, India and the Democratic Republic of Congo) that populate half of the world’s SCD individuals, including other reports from Ghana and Mali [50–53]. Blood group O children (41.4%) dominated, followed by A (29.6%), B (23.3%) and AB (5.7%). The results from our present study agreed with findings from Uganda [54], Cameroon [55], Brazil [56] and Ghana [57]. The G6PD genotypes did not influence the anaemic status at the end of the MNP intervention even though G6PD-deficient RBCs are known to be more vulnerable to oxidative stress, which prevents malaria parasitization and eventually reduces anaemia [58]. Our observation also showed that G6PD alleles were not associated with anaemia in iron- or noniron-containing MNP participants, and these results were similar to reports from countries such as Tanzania [59], Ghana [60] and Nigeria [46].

The iron group HbAS and noniron group HbAC children were less protected against anaemia at the end of the MNP intervention. However, studies have shown that the presence of HbS alleles enhances the removal of parasitized sickle trait red blood cells (RBCs) via the spleen and reduces the cytoadhesion of parasitized erythrocytes, resulting in increased anaemic status among affected haemoglobin genotypes [61]. In agreement with our study, Kreuels et al*.* [51] demonstrated that HbC alleles reduce the cytoadhesion of parasitized erythrocytes, leading to less protection against anaemia [51]. While poorly understood, the reasons for our observation with regard to defending against anaemia for noniron HbAC can be due to reduced parasitization of malaria or other immune mechanisms [51]. Furthermore, the overall Hb and anaemia levels among the participant with malaria were lower compared with those without any malaria infection. However, our study shows that haemoglobin genotypes were not associated with Hb or anaemia induced malaria in MNP children with or without iron. These findings were consistent with the results of the iron supplementation cohort observational study among anaemic Gambian children [62] and Patel et al*.* [63], a study in India. In contrast, Kreuels and his colleagues, in a cohort study, stated that Ghanaian children with HbAS alleles were protected from anaemia [51]. Albiti and Nsiah (2014) also showed that Yemeni children with HbAS alleles were vulnerable to mild anaemia (10.0–10.9 g/dL) but resistant to moderate and severe anaemia [64].

The O blood group has anti-A and B antibodies with no terminal saccharide moiety, making its RBCs more haemolysis-stable than the non-O blood group [65]. However, in our trial, the ABO blood group did not show any defense against anaemia, and this finding corroborates other studies among Nigerian pregnant women [66] and Indians [67]. Blood group O susceptibility to anaemia may be attributable to the pathogenesis of malaria, gender or other unknown causes. Nevertheless, our study failed to establish the link between the ABO blood group among children with iron or noniron-containing MNP and anaemia, as postulated in other findings in India [68] and the case–control analysis in Pakistan [69].

This study was performed during the rainy season (March to September), and malaria parasitaemia was high in both groups. Although malaria parasitaemia was higher in the noniron group than in the iron group, there were similarities between the groups. However, there was a higher risk of malaria for children with G6PD A-A- in both groups. These findings were inconsistent with investigations by Ouattara et al*.* (2014), who reported the existence of a protective association for the G6PD A- variant against uncomplicated malaria in Burkina Faso [70], and a study by Sirugo et al*.* [71], who established heterozygote G6PD A- variant females have immunity against malaria among Gambians [71]. However, our findings are consistent with those of Carter and his colleagues, who found that G6PD status had no association with malaria in six African countries [43]. Moreover, a multi-centre trial involving Kenyan, Gabonese and Ghanaian severe malaria children aged between 6 and 120 months also reported no association between G6PD variants and malaria [72]. The reason for this could be that G6PD does not play any vital antioxidative role under the influence of MNP intervention.

Moreover, our study found that the iron group with HbAS and HbAC was less protected against malaria because the administration of iron increased haemolysis of AS red blood cells by promoting auto-oxidation of haemoglobin S (ferroptosis) and the generation of free oxygen radicals (due to failure of glutathione-dependent antioxidant defences), which damaged the red cell membrane and further damaged it by the deposition of ferritin-like and haemosiderin-like iron on the red cell surface and eventually excessive loss of erythrocytes. Our study was consistent with similar research among pregnant Gambian women with HbAS who had a lower anaemia status when offered iron supplements [73]. However, these findings were in contrast with the concept that heterozygote sickle haemoglobin traits have an advantage in protecting against malaria [51, 64, 74–76]. Similarly, these selective sickle malaria-infected RBCs enhance their removal via the spleen, timely phagocytosis, decrease RBC invasion, reduce parasitization in the venous microvessels due to oxygen stress, and reduce erythrocyte rosetting and cytoadherence [51, 77]. These mechanisms enhance acquired and innate immunity to offer protection against malaria.

Furthermore, blood group A and O participants were less protected against malaria in the Fe group. The possible reason for this could be that malaria parasites easily mimic antigen A, with the erythrocytes of blood group A forming larger and stronger rosettes that selectively allow merozoites to prefer A antigen receptors for RBC invasion. Therefore, the presence of iron acts as a nutrient for the parasites, thereby increasing parasitization and the rapid expression of surface cell membrane proteins to enhance rosette formation and cytoadherence in the endothelia of the microvessels. In addition, iron elevated macrophage-facilitated phagocytes of malaria parasitized RBCs of blood group O, which increased their susceptibility to malaria.

## Conclusion

Iron supplementation increased anaemia in children with haemoglobin AS genotypes and offered less protection against malaria for children with haemoglobin AC and AS and blood groups A and O. Therefore, this new evidence will inform global and national policy on the risks and benefits of iron interventions and guide the implementation of programs to prevent and treat iron deficiency disorders in malaria endemic regions.

## Data Availability

The datasets supporting the conclusions of this article are available; https://doi.org/10.6084/m9.figshare.19768321.v1

## Abbreviations

MNP: Micronutrient powder
Pf: *Plasmodium falciparum*
G6PDD: Glucose-6-phosphate dehydrogenase deficiency
HbAS: Haemoglobin AS
HbAA: Haemoglobin AA
HbAC: Haemoglobin AC
HbCC: Haemoglobin CC
HbSS: Haemoglobin SS
Fe: Iron
Non-Fe: Noniron
IDA: Iron deficiency anaemia
RCT: Randomized controlled trial
WHO: World Health Organization INACG—International Nutritional Anemia Consultative Group
UNICEF: United Nations Children's Fund
GIS: Geographical Information System
BL: Baseline
EL: Endline
RDT: Rapid Diagnostic Test
RFLP: Restriction fragment length polymorphism
EDTA: Ethylene diamine tetra-acetic acid
dNTPs: Deoxynucleotide triphosphates
FBCs: Full blood counts
